# A novel 16-gene alternative mRNA splicing signature predicts tumor relapse and indicates immune activity in stage I–III hepatocellular carcinoma

**DOI:** 10.3389/fphar.2022.939912

**Published:** 2022-09-06

**Authors:** Xu-Xiao Chen, Bao-Hua Zhang, Yan-Cen Lu, Zi-Qiang Li, Cong-Yan Chen, Yu-Chen Yang, Yong-Jun Chen, Di Ma

**Affiliations:** ^1^ Department of General Surgery, Hepatobiliary Surgery, Shanghai Institute of Digestive Surgery, Ruijin Hospital, Shanghai Jiao Tong University School of Medicine, Shanghai, China; ^2^ Department of Clinical Laboratory Medicine, Shanghai Tenth People’s Hospital of Tongji University, Shanghai, China; ^3^ Department of Infectious Diseases, Ruijin Hospital, Shanghai Jiao Tong University School of Medicine, Shanghai, China

**Keywords:** hepatocellular carcinoma, alternative splicing, tumor relapse, prognosis, immunity

## Abstract

**Background:** Hepatocellular carcinoma (HCC) is a lethal disease with high relapse and dismal survival rates. Alternative splicing (AS) plays a crucial role in tumor progression. Herein, we aim to integratedly analyze the relapse-associated AS events and construct a signature predicting tumor relapse in stage I–III HCC.

**Methods:** AS events of stage I–III HCC with tumor relapse or long-term relapse-free survival were profiled to identify the relapse-associated AS events. A splicing network was set up to analyze the correlation between the relapse-associated AS events and splicing factors. Cox regression analysis and receiver operating characteristic curve were performed to develop and validate the relapse-predictive AS signature. Single-sample gene set enrichment analysis (ssGSEA) and the ESTIMATE algorithm were used to assess the immune infiltration status of the HCC microenvironment between different risk subgroups. Unsupervised cluster analysis was conducted to assess the relationship between molecular subtypes and local immune status and clinicopathological features.

**Results:** In total, 2441 ASs derived from 1634 mRNA were identified as relapse-associated AS events. By analyzing the proteins involved in the relapse-associated AS events, 1573 proteins with 11590 interactions were included in the protein–protein interaction (PPI) network. In total, 16 splicing factors and 61 relapse-associated AS events with 85 interactions were involved in the splicing network. The relevant genes involved in the PPI network and splicing network were also analyzed by Gene Ontology enrichment analysis. Finally, we established a robust 16-gene AS signature for predicting tumor relapse in stage I–III HCC with considerable AUC values in all of the training cohort, testing cohort, and entire cohort. The ssGSEA and ESTIMATE analyses showed that the AS signature was significantly associated with the immune status of the HCC microenvironment. Moreover, four molecular subgroups with distinguishing tumor relapse modes and local immune status were also revealed.

**Conclusion**: Our study built a novel 16-gene AS signature that robustly predicts tumor relapse and indicates immune activity in stage I–III HCC, which may facilitate the deep mining of the mechanisms associated with tumor relapse and tumor immunity and the development of novel individualized treatment targets for HCC.

## Introduction

Liver cancer is the third leading cause of cancer-associated deaths worldwide (Sung et al., 2021). Hepatocellular carcinoma (HCC) is the most predominant type of liver cancer, accounting for 75–85% of cases (Singal et al., 2020). Surgical resection remains the most effective therapy for HCC with curative potential, yet the high frequency of tumor relapse (50–70% for 5 years after surgery) hinders the improved survival (European Association for the Study of the Liver Electronic address and European Association for the Study of the Liver, 2018). Relevant data revealed that 70% and more than 90% of the tumor relapse occurred within 2 and 5 years after surgery, respectively (Zheng et al., 2017). Tumor relapse of HCC is always associated with poor therapeutic response and survival due to aggressive pathological characteristics. Hence, a thorough exploration of the mechanisms underlying tumor relapse and discovery of robust relapse predictive factors for HCC is urgently needed to further improve the long-term prognosis of HCC patients.

Tumor progression is a complex process involving many genetic alterations, which may result in activating oncogenes, inactivating tumor suppressors, or enhancing tumor cells invading normal organs/tissues. The alterations in the genetic central dogma lead to aberrant expression of relevant oncogenes or suppressor genes. Alternative splicing (AS) of mRNA is a common event in the process of the genetic central dogma, which occurs in more than 95% of human multi-exon genes and results in encoding of different splicing and protein isoforms (Nilsen and Graveley, 2010). In addition to that, the translation of mRNA isomer can be downregulated by termination codons originating from AS switches (Climente-Gonzalez et al., 2017). Therefore, AS plays a critical role in the maintenance of homeostasis for cells or organisms, and increasing evidence reveals that dysregulation of AS is closely inclined to various diseases, including tumor development, immune escape, and progression (Urbanski et al., 2018; Zhang et al., 2021a). AS events are regulated by splicing factors (SFs); changed expression or mutations of SFs can lead to complete alterations of AS and may result in tumor-specific splicing isoforms in human cancers (Salton et al., 2015; Wang et al., 2019; Song et al., 2020). Thus, dissecting the tumor-specific AS isoforms and the splicing network between ASs and SFs could provide insights into the mechanisms of tumor development and progression, which may offer prognostic tumor biomarkers and potential therapeutic targets.

Several studies have profiled the tumor-specific AS events in HCC and identified AS signatures that are associated with overall survival in HCC (Zhu et al., 2019; Cai et al., 2020; Wu et al., 2020). However, a comprehensive depiction of the relapse-associated AS events and robust AS signatures predicting tumor relapse in HCC remains lacking. Herein, we integratedly analyze the genome-wide AS events from the HCC cohort in The Cancer Genome Atlas (TCGA) database and illustrate the relapse-associated AS events in stage I–III HCC. More importantly, we built a novel 16-gene AS signature that predicts tumor relapse and indicates immune activity in stage I–III HCC with high performance, shedding light on the individualized therapeutic targets for HCC.

## Materials and Methods

### Data extraction and pre-processing

**FIGURE 1:**
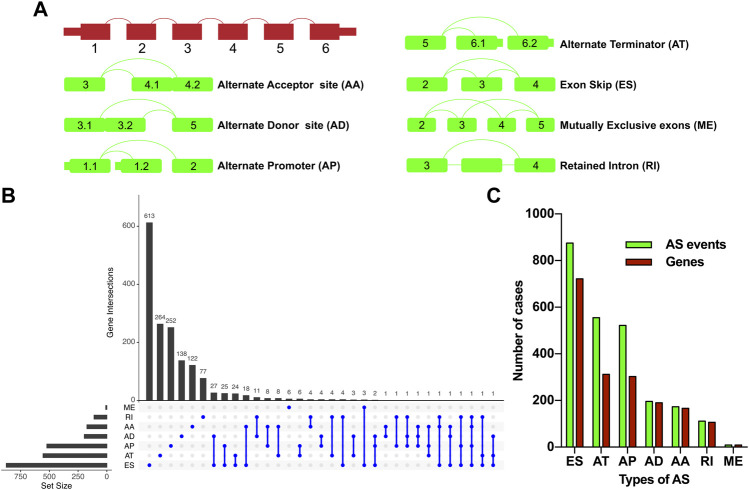
Overview of the relapse-associated AS event profiling in HCC. **(A)** Illustrations for the seven subtypes of AS events including AA, AD, AP, AT, ES, ME, and RI. **(B)** UpSet plot delineating the overlaps of the seven subtype AS events and the relevant mRNA. One mRNA owns four splicing patterns at the maximum. **(C)** Number of the seven subtype AS events and the involved genes. Abbreviations: AA, alternate acceptor site; AD, alternate donor site; AP, alternate promoter; AT, alternate terminator; ES, exon skip; ME, mutually exclusive exons; RI, retained intron.

Raw RNA sequence data of the liver hepatocellular carcinoma (LIHC) cohort was extracted from the TCGA database (https://portal.gdc.cancer.gov/) (Hutter and Zenklusen, 2018), and the corresponding clinicopathological information including age, sex, hepatitis virus infection status, tumor grade, tumor stage, relapse, and survival status was downloaded from the University of California Santa Cruz (UCSC) Xena platform (https://xena.ucsc.edu/) (Goldman et al., 2020). The inclusion criteria for the present study were as follows: (I) R0 resection was achieved in the surgical procedure; (II) the histopathological diagnosis was HCC; (III) the pathological TNM stage of HCC was stage I, stage II, or stage III; (IV) HCC patients with complete clinicopathological and survival information; (V) HCC patients with overall survival time over than 30 days; (VI) corresponding AS event data were available. According to the inclusion criteria, we finally enrolled 277 HCC patients in this study for further analysis. The mRNA AS profiles of HCC were obtained from TCGA SpliceSeq (https://bioinformatics.mdanderson.org/TCGA-SpliceSeq/) (Ryan et al., 2016). Seven types of AS were illustrated in Figure 1A, namely, alternate acceptor site (AA), alternate donor site (AD), alternate promoter (AP), alternate terminator (AT), exon skip (ES), mutually exclusive exons (ME), and retained intron (RI). The percent-spliced-in (PSI) value, ranging from 0 to 1, was a normalized method to evaluate the AS events. In order to achieve credible data on AS events, the percentage of clinical samples with PSI values greater than or equal to 75% were included in the present study. Furthermore, relevant splicing factor (SF) data were acquired from the SpliceAid-F database (http://www.caspur.it/SpliceAidF) (Giulietti et al., 2013).

### Identification of relapse-associated alternative splicing events in stage I–III hepatocellular carcinoma

The cohort of enrolled stage I–III HCC patients was divided into a relapse group and a long-term relapse-free survival (RFS) group. The relapse group was defined as HCC patients who suffered tumor recurrence or distant metastasis after R0 hepatic resection, and the long-term RFS group was defined as HCC patients without tumor recurrence or distant metastasis after a minimum follow-up time of 3 years after R0 hepatic resection. The propensity score matching (PSM) method was conducted to achieve more balanced groups by matching TNM stage and Path_T, which showed a significant influence on tumor relapse. The relapse group and long-term RFS group were matched as 2:1. The Wilcoxon test was performed to identify relapse-associated AS events, and *p-*values less than 0.05 were considered statistically significant. The UpSet plot was used for illustrating the relapse-associated AS events. Gene Ontology (GO) functional enrichment analysis was performed using the R package clusterProfiler on the relevant genes of the relapse-associated AS events to identify significantly enriched biological processes (BP), cellular components (CC), and molecular functions (MF).

### Construction of protein–protein interaction network and regulatory splicing network

The related genes based on the relapse-associated AS events were further analyzed by performing protein–protein interaction (PPI) analysis on the online STRING Version 11.5 database (www.string-db.org/) (Szklarczyk et al., 2019). MCODE and Cytohubba in Cytoscape (version 3.8.2) were used for clustering the PPI network and selecting hub proteins, respectively. The correlation between the expression level of SFs and the PSI level of the relapse-associated AS events was assessed by Spearman’s test; *p* values less than 0.01 and the values of correlation coefficient (cor) less than −0.55 or over 0.55 were considered statistically significant. Finally, the results of the PPI network and the regulatory splicing network were visualized by Cytoscape (version 3.8.2).

### Survival analysis and alternative splicing signature identification for predicting tumor relapse in stage I–III hepatocellular carcinoma

The enrolled stage I–III HCC patients were randomly divided into the training cohort and testing cohort with a ratio of 7:3. The training cohort was used to build the relapse predictive AS signature, while the testing cohort and entire cohort were used to validate the accuracy. Then, a univariate Cox regression analysis was performed to explore the effects of the identified relapse-associated AS events on RFS in stage I–III HCC, and *p* values less than 0.05 were considered statistically significant. The data of those AS events were visualized as volcano plot, UpSet plot, and bubble plot. To enhance the robustness of the signature, only AS events with *p* values less than 0.0005 in the univariate Cox regression model were included for further screening by LASSO Cox regression analysis (Liu et al., 2021a; Zhang et al., 2021b). Finally, multivariate Cox regression analysis was conducted, and AS events with *p* values less than 0.05 were selected to establish the relapse predictive signature. The predictive accuracy of the final signature was evaluated by risk score analysis, RFS survival analysis, and receiver operating characteristic (ROC) curve. Subgroup analysis was also conducted to further investigate the prognostic significance of the signature *via* stratifying the entire cohort into different subgroups based on age, gender, T stage, and TNM stage. The cutoff point of the low-risk and high-risk score group was identified using the R package survminer. The univariate, LASSO, and multivariate Cox regression analyses were conducted using R language (version 4.0.3).

### Functional enrichment analysis and immune activity analysis

Kyoto Encyclopedia of Genes and Genomes (KEGG) functional enrichment analysis was performed using the R package clusterProfiler to identify the biological functions and pathways associated with the relapse predictive AS signature. Single-sample gene set enrichment analysis (ssGSEA) was conducted to analyze the local immune infiltration levels of immune cell types, immune-related functions, and pathways in HCC using the R package GSVA (Liu et al., 2021b; Wang et al., 2022). Moreover, the ESTIMATE algorithm was applied using the R package ESTIMATE to evaluate the infiltration degrees of immune and stromal cells within the HCC microenvironment, which further validated the effectuality of ssGSEA analysis.

### Identification and analysis of molecular subtype clusters

Considering AS events varied much differently at the personal level, unsupervised consensus clustering was performed by the R package ConsensusClusterPlus to achieve a more robust classification based on the AS events involved in the relapse predictive signature. Elbow and Gap analysis was conducted to identify the optional number of clusters, and the consensus molecular subtype was classified using the R package CMScaller. The correlation between molecular subtypes and RFS was illustrated as the Kaplan–Meier plot, and the correlations between molecular subtypes and local immune infiltration status and clinicopathological features were illustrated as a heatmap.

## Results

### Identification of relapse-associated alternative splicing events in stage I–III hepatocellular carcinoma

277 patients with stage I–III HCC from the TCGA-LIHC cohort were enrolled in the present study; 131 cases and 39 cases of which were sorted as the relapse group and long-term RFS group, respectively. PSM analysis was conducted to achieve more balanced groups by matching TNM stage and Path_T, which showed a significant influence on tumor relapse. The summary clinicopathological features of the two groups before and after PSM are listed in Table 1. Seven types of AS, namely, AA, AD, AP, AT, ES, ME, and RI are presented in Figure 1A. To identify the AS events associated with HCC relapse, we compared the PSI values of the HCC patients in the two groups. Then, we identified 2,441 significantly differently expressed AS events derived from 1,634 mRNA, containing 875 ES from 722 mRNA, 555 AT from 312 mRNA, 522 AP from 303 mRNA, 196 AD from 190 mRNA, 173 AA from 166 mRNA, 111 RI from 106 mRNA, and nine ME from nine mRNA (Figure 1C). The most frequent AS type was ES which accounted for more than half of the total relapse-associated AS events, while ME was the least common AS type. The overview of these AS events was depicted in the UpSet plot, which also presented the overlapping of the relapse-associated AS events and relevant mRNA in detail (Figure 1B). As illustrated in the UpSet plot, diverse types of AS events might be derived from one mRNA, and one single mRNA could possess four splicing patterns at the maximum. To investigate the potential function of the relapse-associated AS events, the relevant genes of which underwent GO enrichment analysis were analyzed. Representative significant enriched terms (*p* < 0.05) in the three GO categories (BP, CC, and MF) are shown in Supplementary Figure S1.

**TABLE 1:** Clinicopathological characteristics of HCC patients in relapse and long-term RFS groups before and after PSM.

Clinicopathological index		Before PSM			After PSM		
		Relapse	Long-term RFS	P	Relapse	Long-term RFS	P
		(*n* = 131)	(*n* = 39)		(*n* = 78)	(*n* = 39)	
Age (year)	<60	65	21	0.779	42	21	1.000
	≥60	66	18		36	18	
Sex	Female	40	16	0.303	28	16	0.736
	Male	91	23		50	23	
Alcohol consumption	No	83	29	0.420	51	29	0.627
	Yes	40	9		23	9	
	NA	8	1		4	1	
Viral hepatitis	Negative	69	27	0.316	48	27	0.774
	HBV	23	5		15	5	
	HCV	4	1		4	1	
	HBV + HCV	35	6		11	6	
Liver cirrhosis	No	54	20	0.197	39	20	0.871
	Yes	35	6		15	6	
	NA	42	13		24	13	
Vascular invasion	No	66	26	0.224	47	26	0.812
	Yes	40	9		20	9	
	NA	25	4		11	4	
Tumor grade	G1	14	7	0.508	12	7	0.897
	G2	59	17		39	17	
	G3	53	13		24	13	
	G4	4	2		3	2	
	NA	1	0		0	0	
TNM stage	I	53	27	0.005	47	27	0.625
	II	35	7		17	7	
	III	43	5		14	5	
Path_T	T1	54	27	0.020	47	27	0.718
	T2	35	7		17	7	
	T3	38	5		14	5	
	T4	4	0		0	0	
Path_N	N0	102	32	0.786	62	32	0.810
	N1	2	0		0	0	
	NX	27	7		16	7	
Path_M	M0	105	33	0.695	64	33	0.931
	MX	26	6		14	6	

### Construction of protein–protein interaction network and regulatory splicing network

**FIGURE 2:**
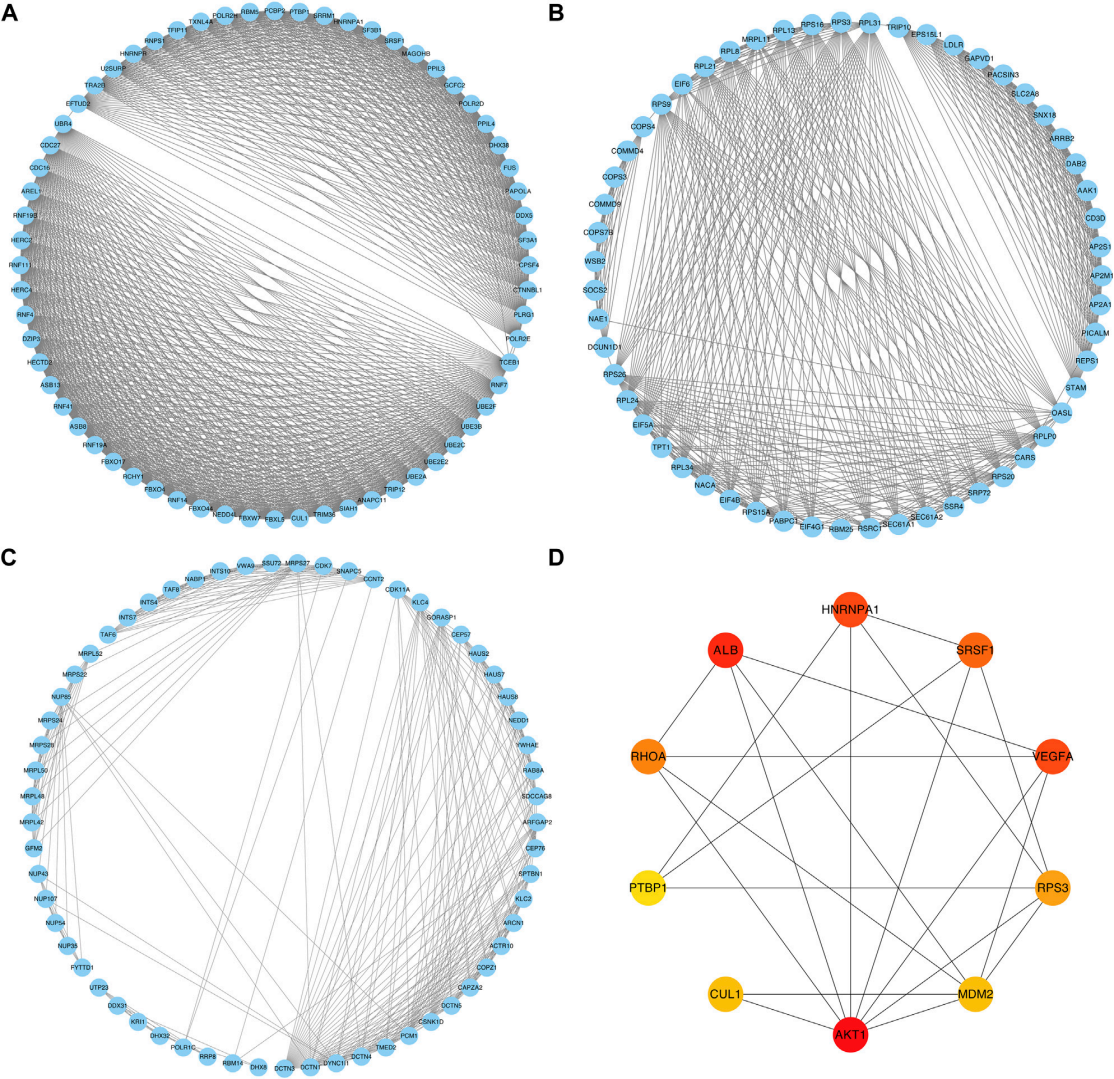
PPI networks of the relevant proteins of the relapse-associated AS events. **(A)** PPI network of the cluster with the highest score. **(B)** PPI network of the cluster ranking second. **(C)** PPI network of the cluster ranking third. **(D)** Network of the top ten hub proteins in the cluster owning the highest score. The color of the nodes represents the rank of hub proteins ordered as follows: red, orange, and yellow.

The PPI network was constructed based on the relevant proteins of the relapse-associated AS events, totally including 1573 proteins with 11590 interactions (Supplementary Table S1). Meanwhile, 34 clusters were identified, and the individual score of those clusters ranged from 2.500 to 31.937. Figures 2A–C in detail depicted the top three clusters, which scored 31.937, 18.222, and 10.367. In the cluster owning the highest score, 64 proteins with 1006 interactions were included (Figure 2A); in the cluster ranking second, 55 proteins with 492 interactions were included (Figure 2B); in the following cluster, 61 proteins with 311 interactions were included (Figure 2C). Among the proteins included in the cluster with the highest score, ten proteins (AKT1, ALB, HNRNPA1, VEGFA, SRSF1, RHOA, RPS3, MDM2, CUL1, and PTBP1) were identified as hub proteins, in which, AKT1 and ALB obtained the top place followed by HNRNPA1, VEGFA, and SRSF1, while PTBP1 was the tail ender (Figure 2D).

**FIGURE 3:**
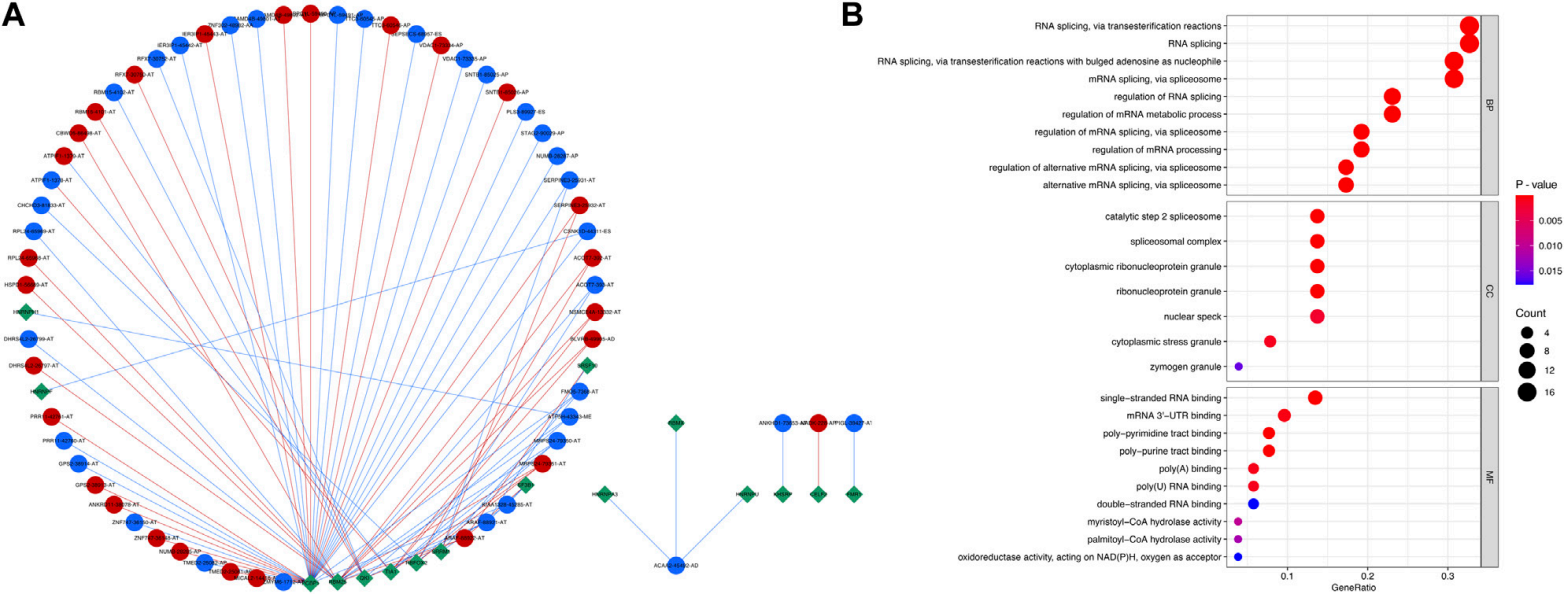
Regulatory splicing network and functional enrichment analysis of the SFs and relapse-associated AS events. **(A)** Regulatory splicing network of the SFs and relapse-associated AS events. Diamond nodes represent SF, and the circle nodes represent AS events. The AS events represented by red nodes and blue nodes were upregulated and downregulated, respectively, by comparison of the relapse group to the long-term RFS group. The red and blue lines showed positive and negative correlations between SFs and AS events, respectively. **(B)** Functional enrichment analysis of the relevant genes involved in the regulatory splicing network. The top ten significant enriched terms in the three GO categories (BP, CC, and MF) were shown. Abbreviations: BP, biological processes; CC, cellular components; GO, Gene Ontology; MF, molecular functions.

A regulatory splicing network was constructed by analyzing the correlation of the expression level of the SFs and the PSI level of the relapse-associated AS events with a strict standard. In total, 16 SFs were significantly related to 60 relapse-associated AS events by forming 85 interactions (including 36 positive and 49 negative regulation) in the splicing network. Moreover, 27 relapse-associated AS events were significantly upregulated and the others were significantly downregulated in the relapse group, as compared with the long-term RFS group. ARAF-88922-AT, ARAF-88921-AT, FMO5-7368-AT, MRPS24-79351-AT, and MRPS24-79350-AT were considered the hub relapse-associated AS events in the splicing network. Meanwhile, PCBP1, RBM25, QKI, TIA1, and RBFOX2 were identified as the core SFs, which owned the most interaction with the relapse-associated AS events, implying their dominant position in determining the relapse-associated AS events in stage I–III HCC (Figure 3A; Supplementary Table S2). Subsequently, the relevant genes involved in the regulatory splicing network underwent GO enrichment analysis. Representative significant enriched terms (*p* < 0.05) in the three GO categories (BP, CC, and MF) are shown in Figure 3B.

### Survival analysis and relapse predictive alternative splicing signature construction

**FIGURE 4:**
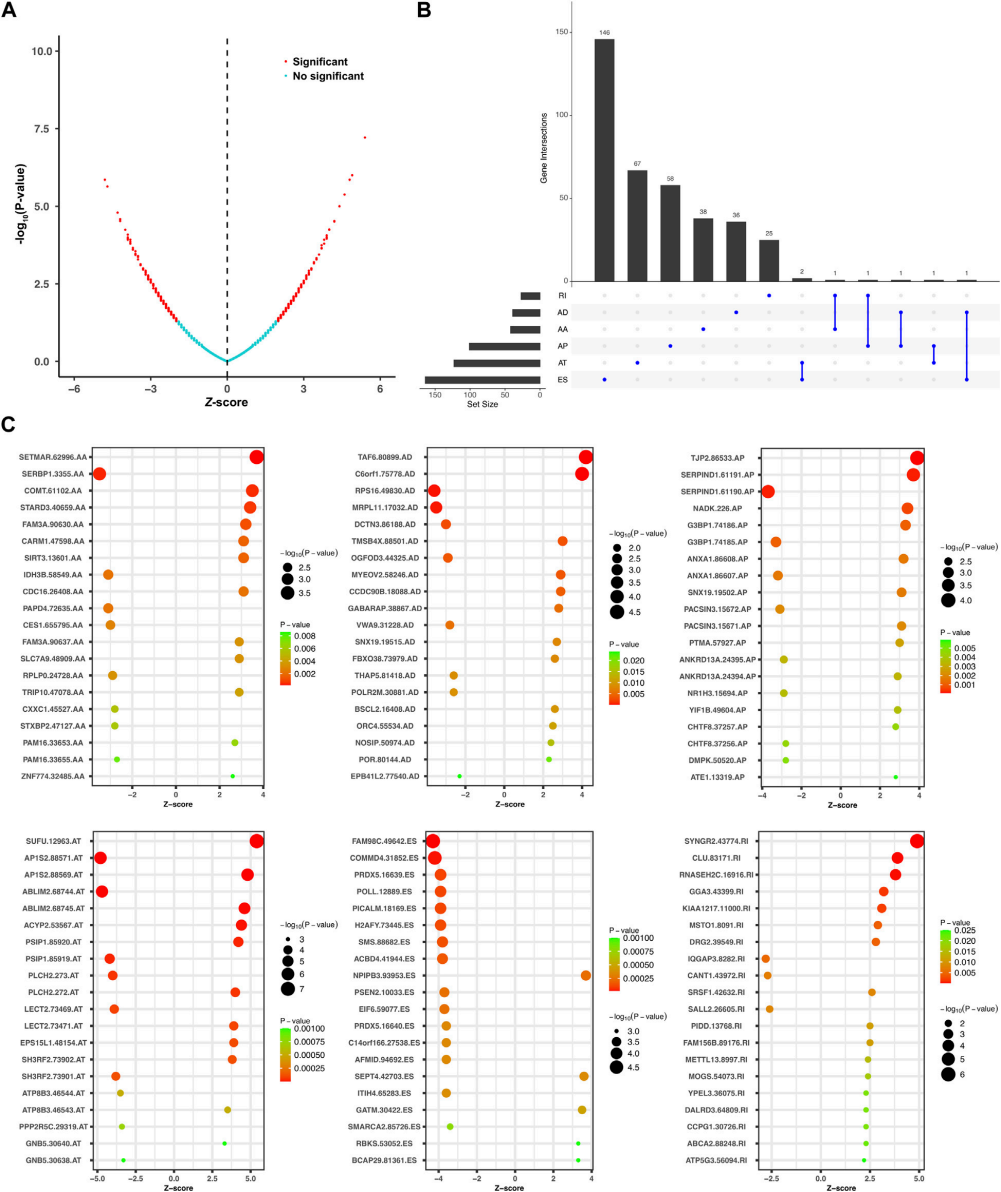
Overview of the relapse-associated AS events from initial screening by univariate Cox regression analysis. **(A)** Volcano plot showing the statistically significant relapse-associated AS events, which was presented by red. **(B)** UpSet plot delineating the overlaps of the statistically significant relapse-associated AS events and the relevant mRNA. One mRNA owns two splicing patterns at the maximum. **(C)** Bubble plots delineating the distribution of each type of statistically significant relapse-associated AS events after univariate Cox regression analysis. Representative top 20 AS events in AA, AD, AP, AT, ES, and RI were shown.

**FIGURE 5:**
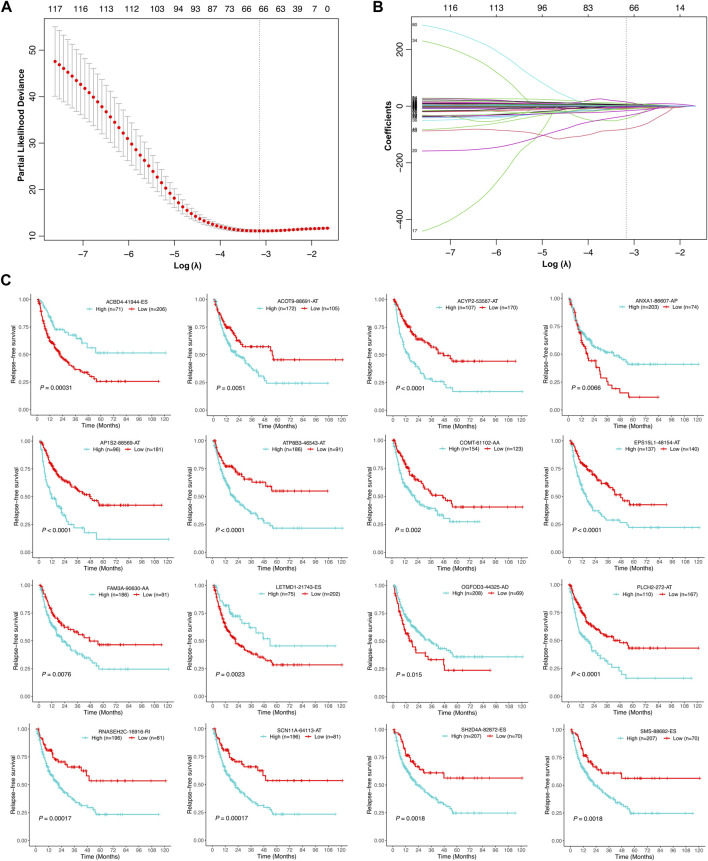
Selection of the optimal relapse-associated AS events for constructing the relapse predictive signature. **(A)** Cross-validation for tuning parameter selection in the LASSO Cox regression. The optimal value determined by minimum criteria is marked by a dotted vertical line. **(B)** LASSO coefficient profiles of the candidate relapse-associated AS events in the LASSO Cox regression. The optimal value determined by minimum criteria is marked by a dotted vertical line. **(C)** Kaplan–Meier survival analysis of the individual AS events involved in the relapse predictive signature.

Univariate Cox regression analysis was further performed to explore the prognostic value of the relapse-associated AS events mentioned earlier. A total of 496 AS events derived from 377 mRNA were detected to be prognostically significant (*p* < 0.05). The statistically significant AS events (Z-score < −2 or > 2, *p* < 0.05) are shown in the Volcano plot and Upset plot (Figures 4A,B). The Upset plot also showed the overlapping of the statistically significant AS events and relevant mRNA in detail (Figure 4B). ME was the only AS type that was not involved in the statistically significant AS events, and ES was still the most frequent AS type. A bubble plot was further performed to depict the representative top 20 AS events in each AS type (Figure 4C). SETMAR-62996-AA, SERBP1-3355-AA, and COMT-61102-AA in AS type of AA; TAF6-80899-AD, C6orf1-75778-AD and RPS16-49830-AD in AS type of AD; TJP2-86533-AP, SERPIND1-61191-AP, and SERPIND1-61190-AP in AS type of AP; SUFU-12963-AT, AP1S2-88571-AT, and AP1S2-88569-AT in AS type of AT; FAM98C.49642.ES, COMMD4-31852-ES, and PRDX5-16639-ES in AS type of ES; SYNGR2-43774-RI, CLU.83171.RI, and RNASEH2C.16916.RI in the AS type of RI were the top three AS events in each AS type. Subsequently, the AS events with *p* values less than 0.0005 in the univariate Cox regression were further screened by LASSO Cox regression analysis. The results of cross-validation for tuning parameter selection and LASSO coefficient profiles of the AS events are shown in Figures 5A,B. Through minimum criteria, 66 AS events with non-zero coefficients at lambda.min were filtered by the LASSO Cox regression analysis.

Finally, those 66 AS events were analyzed by multivariate Cox regression analysis. AS events with *p* values less than 0.05 were selected to establish the relapse predictive signature, and 16 AS events were included (Table 2). Kaplan–Meier survival analysis revealed that all the 16 AS events were significantly associated with the RFS of HCC patients; high expression of ACOT9-88691-AT, ACYP2-53567-AT, AP1S2-88569-AT, ATP8B3-46543-AT, COMT-61102-AA, EPS15L1-48154-AT, FAM3A-90630-AA, PLCH2-272-AT, RNASEH2C-16916-RI, SCN11A-64113-AT, SH2D4A-82872-ES, and SMS-88682-ES was correlated with poor RFS in HCC, whereas low expression of ACBD4-41944-ES, ANXA1-86607-AP, LETMD1-21743-ES, and OGFOD3-44325-AD was correlated with poor RFS in HCC (Figure 5C). Based on the 16-gene AS signature, the following formula was developed to calculate the risk score of tumor relapse: risk score = (−9.15 × PSI value of ACBD4-41944-ES) + (6.14 × PSI value of ACOT9-88691-AT) + (12.70 × PSI value of ACYP2-53567-AT) + (−41.19 × PSI value of ANXA1-86607-AP) + (3.01 × PSI value of AP1S2-88569-AT) + (2.65 × PSI value of ATP8B3-46543-AT) + (20.94 × PSI value of COMT-61102-AA) + (2.98 × PSI value of EPS15L1-48154-AT) + (4.98 × PSI value of FAM3A-90630-AA) + (−1.48 × PSI value of LETMD1-21743-ES) + (−6.67 × PSI value of OGFOD3-44325-AD) + (1.65 × PSI value of PLCH2-272-AT) + (3.77 × PSI value of RNASEH2C-16916-RI) + (9.59 × PSI value of SCN11A-64113-AT) + (8.13 × PSI value of SH2D4A-82872-ES) + (−80.63 × PSI value of SMS-88682-ES).

**TABLE 2:** Information of ASs included in the signature.

Gene	As id	Splice type	Exon	From exon	To exon
ACBD4	41944	ES	10	9.2	12
ACOT9	88691	AT	13.2	NA	NA
ACYP2	53567	AT	3	NA	NA
ANXA1	86607	AP	1	NA	NA
AP1S2	88569	AT	5.2	NA	NA
ATP8B3	46543	AT	14.2	NA	NA
COMT	61102	AA	6.1	4	6.2
EPS15L1	48154	AT	23.2	NA	NA
FAM3A	90630	AA	7.1	6	7.2
LETMD1	21743	ES	3.3:4:5:6	3.2	7
OGFOD3	44325	AD	2.2	2.1	3
PLCH2	272	AT	23.2	NA	NA
RNASEH2C	16916	RI	1.2	1.1	1.3
SCN11A	64113	AT	26	NA	NA
SH2D4A	82872	ES	3	1	4
SMS	88682	ES	3	2	4

Abbreviations: NA, not available.

**FIGURE 6:**
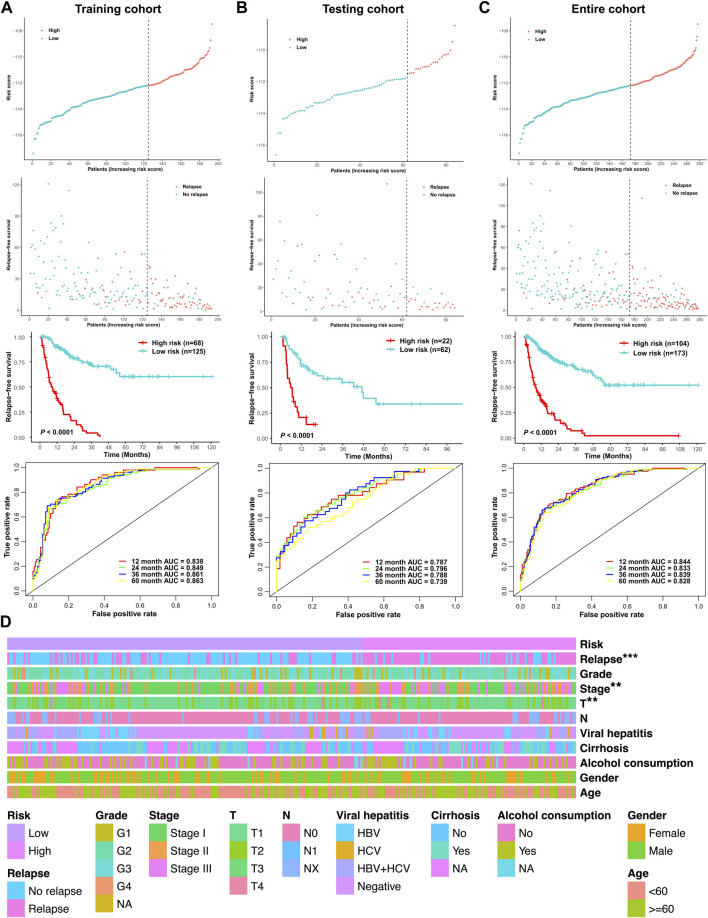
Assessment of the efficacy of the signature in predicting tumor relapse in HCC. **(A)** Efficacy of the relapse predictive signature in the training cohort. **(B)** Efficacy of the relapse predictive signature in the testing cohort. **(C)** Efficacy of the relapse predictive signature in the entire cohort. The upper panel depicted the risk score analysis, the middle panel depicted the Kaplan–Meier survival analysis, and the lower panel depicted the ROC curve analysis. **(D)** Heatmap of the relationship between the risk score and the clinicopathological features in the entire cohort. **p < 0.01 and ***p < 0.001.

The cutoff point of the risk score was determined using the R package survminer. According to the cutoff point, the three cohorts of the stage I–III HCC patients (the training cohort, testing cohort, and entire cohort) were divided into the low-risk group and high-risk group, respectively. Risk score and RFS survival analysis suggested that the relapse predictive signature has great efficiency in distinguishing the low-risk and high-risk groups in all three cohorts (Figures 6A–C). To further assess the performance of the relapse predictive signature, the ROC curve was plotted at different time points after surgery. The AUC values of different ROC curves at 1, 2, 3, and 5 year after surgery were considerable and stable in all three cohorts, which displayed good relapse predictive performance (Figures 6A–C). The relationship between the risk score and the clinicopathological features in the entire cohort was illustrated as a heatmap, and significant correlations were identified between the risk score and tumor relapse, tumor stage, and T classification (Figure 6D). Furthermore, univariate and multivariate Cox regression analysis was performed to assess the significance of the clinicopathological characteristics and risk score. The results showed that the risk score was an independent predictor of tumor relapse in HCC patients (Supplementary Figure S2).

**FIGURE 7:**
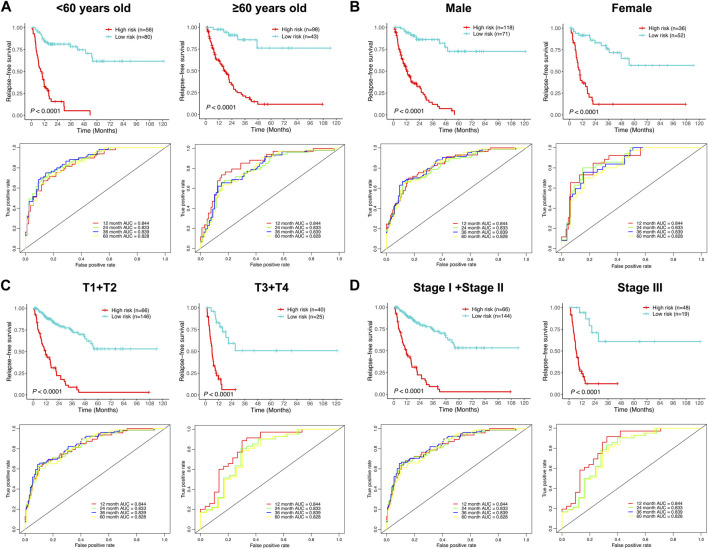
Assessment of the efficacy of the signature in diverse subgroups with different clinicopathological features. **(A)** Age (< 60 and ≥ 60). **(B)** Gender (male and female). **(C)** T stage (T1 + T2 and T3 + T4). **(D)** TNM stage (stage I + stage II and stage III). The upper panel depicted the Kaplan–Meier survival analysis, and the lower panel depicted the ROC curve analysis.

To further investigate the prognostic significance of the relapse predictive signature in HCC, subgroup analysis was conducted *via* stratifying the entire cohort into different subgroups according to age (< 60 and ≥ 60), gender (male and female), T stage (T1 + T2 and T3 + T4), and TNM stage (stage I + stage II and stage III). The results of Kaplan–Meier survival analysis and ROC curve analysis showed that the relapse predictive signature was stable and had a great performance in different subgroups (Figures 7A–D).

### Functional enrichment analysis and immune activity analysis based on the risk mode

**FIGURE 8:**
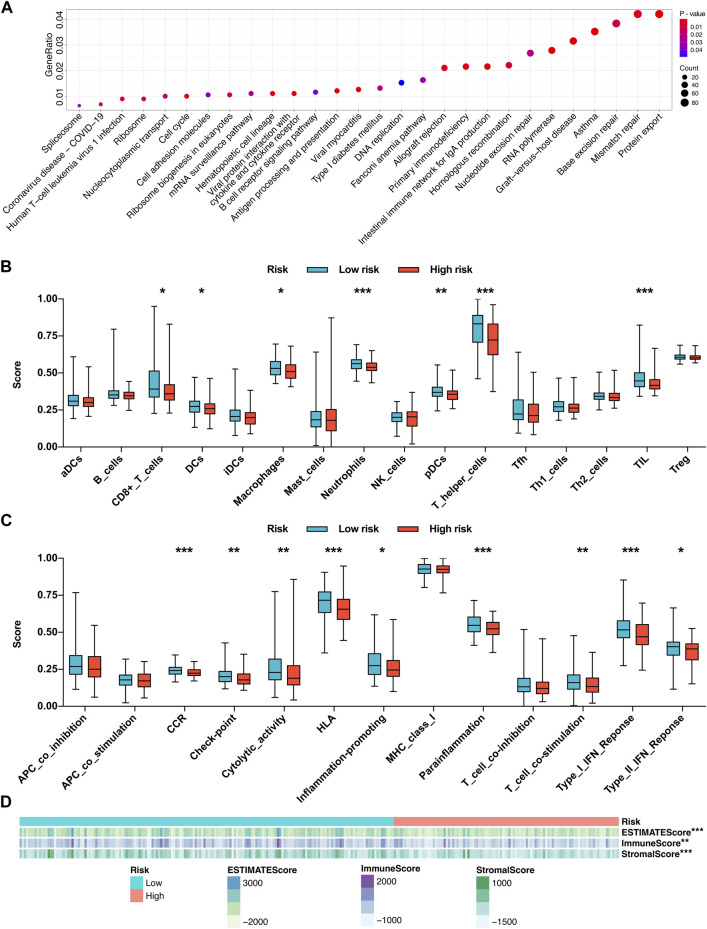
Functional enrichment analysis and immune activity analysis based on the risk mode. **(A)** KEGG functional enrichment analysis of the differentially expressed genes between the two risk subgroups. **(B)** Infiltration fractions of 16 immune cell types in the two risk subgroups. **(C)** Comparisons of 13 immune-related functions in the two risk subgroups. **(D)** Heatmap of the relationship between the risk score and the infiltration degrees of immune and stromal cells within the tumor microenvironment. *p < 0.05, **p < 0.01, and ***p < 0.001.

Due to the different tumor relapse patterns of HCC patients in the low-risk group and high-risk group, differentially expressed genes between the two risk subgroups were explored and subjected to KEGG functional enrichment analysis. The KEGG pathway analysis revealed that several immune-related pathways were significantly enriched, such as graft-versus-host disease, intestinal immune network for IgA production, primary immunodeficiency, allograft rejection, antigen processing and presentation, B-cell receptor signaling pathway, viral protein interaction with cytokine and cytokine receptor, cell adhesion molecules, and human T-cell leukemia virus 1 infection (Figure 8A).

Driven by the results of KEGG pathway analysis, we further investigated the local immune characteristics of the two risk subgroups using ssGSEA analysis, and the enrichment scores of ssGSEA for 16 immune cells and 13 immune-related functions or pathways between the two risk subgroups were compared. The low-risk group showed higher local infiltration fractions of several immune cell types, including CD8^+^ T cells, dendritic cells (DCs), macrophages, neutrophils, plasmacytoid DCs (pDCs), T helper (Th) cells, and tumor-infiltrating lymphocytes (TILs) (Figure 8B). Similarly, the local immune functions of chemokine receptors (CCR), check point, cytolytic activity, human lymphocyte histocompatibility antigen (HLA), inflammation-promoting, parainflammation, T cell co-stimulation, type I interferon (IFN) response, type II IFN response were also more activated in the low-risk group (Figure 8C). Moreover, the ESTIMATE algorithm was applied to evaluate the infiltration degrees of immune and stromal cells within the tumor microenvironment. The results revealed that the low-risk group exhibited significantly higher ESTIMATE score, immune score, and stromal score, which further confirmed the ssGSEA results (Figure 8D). Conclusively, those findings suggested that the local immune activity within the HCC microenvironment, which may benefit the antitumor effects, is more activated in the low-risk group.

### Molecular subtype clusters associated with tumor relapse and local immune status

**FIGURE 9:**
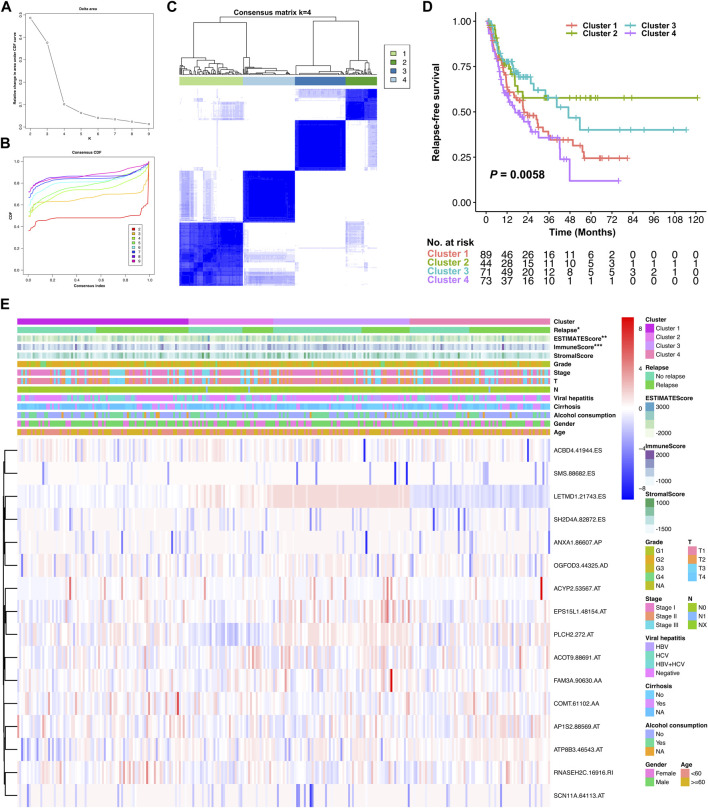
Identification of relapse-associated AS clusters related to clinical outcomes and molecular subtypes. **(A)** Elbow analysis for identifying the optimal number of clusters. **(B)** Gap analysis for identifying the optimal number of clusters. **(C)** Consensus clustering matrix depicting the four clusters of the patients. **(D)** Kaplan–Meier survival analysis of the four clusters of HCC patients. **(E)** Heatmap of the relationship of the four molecular subtype clusters and the local immune infiltration status, clinicopathological characteristics. *p < 0.05, **p < 0.01, and ***p < 0.001.

Unsupervised consensus clustering was further conducted for all the stage I–III HCC patients, based on the AS events involved in the relapse predictive signature. Elbow and Gap analysis showed that the optimal number of clusters was four groups (Figures 9A,B). Then, the four clusters were defined by the distribution of the consensus value of each sample, which was classified as follows: cluster 1 (*n* = 89, 32.1%), cluster 2 (*n* = 44, 15.9%), cluster 3 (*n* = 71, 25.6%), and cluster 4 (*n* = 73, 26.4%) (Figure 9C). The Kaplan–Meier curve was plotted to evaluate the association between clustering and RFS. The results revealed that different molecular subtype clusters were related with diverse RFS patterns as shown in Figure 9D. Cluster 2 had the best RFS of the four clusters followed by cluster 3, while cluster 1 and cluster 4 showed relatively poor outcomes in the RFS analysis. Finally, the association of the four molecular subtype clusters and the local immune infiltration status and clinicopathological features was illustrated as a heatmap, and significant correlations were identified between the molecular subtype cluster and tumor relapse, ESTIMATE score, and immune score (Figure 9E). Collectively, these data clearly indicated that the molecular subtype clustering based on the relapse predictive signature has a good performance in distinguishing the different relapse patterns and local immune infiltration status between diverse clusters.

## Discussion

Despite the great progress in surveillance and therapeutic strategies improving overall survival, the clinical outcomes of HCC remain dismal due to high frequency of tumor relapse, even after curative surgery (Bosch et al., 2004; Rahbari et al., 2011). With HCC relapse, the therapeutic options are limited and the treatment response is usually poor due to the aggressive pathological characteristics, which certainly leads to poor prognosis. Early precise warning and detection of HCC with a high risk of tumor relapse may be the optimal maneuver to settle this issue. Until now, the TNM staging system of the American Joint Committee on Cancer (AJCC), together with other prognostic staging systems (BCLC, CLIP, and JIS classification of HCC), is most commonly used to assess the prognosis of HCC (Pons et al., 2005; Amin et al., 2017). However, these prognostic staging systems are mostly focused on clinical features but ignore the genetic and epigenetic dysregulations in the process of HCC development and progression, which always makes them not sufficient for efficiently predicting tumor relapse or prognosis of HCC. Development of robust signatures based on the molecular biological process in HCC to predict tumor relapse would be probable to supplement the current prognostic staging systems and guide the therapeutic strategies after surgery, further improving the prognosis of HCC patients. AS is an important biological process and has been demonstrated to play a critical role in the genetic central dogma (Nilsen and Graveley, 2010; Kozlovski et al., 2017). Dysregulation of AS is closely inclined to tumor development, immune escape, drug resistance, and progression (Sciarrillo et al., 2020; Zhang et al., 2021a; Su and Huang, 2021). However, there is little literature dissecting the mechanism of AS in tumor relapse of HCC, and a comprehensive depiction of the relapse-associated AS events and robust AS signatures predicting tumor relapse in HCC remains lacking.

In the present study, we focused on stage I–III HCC received curative resection and comprehensively analyzed the relapse-associated AS events in these cases. In total, 2441 ASs derived from 1634 mRNA were identified as relapse-associated AS events. ES was determined as the most common AS event among the seven AS subtypes, implying its critical role in the tumor relapse of HCC. In the PPI network based on the relevant proteins of these AS events, 34 clusters including 1573 proteins with 11590 interactions were identified. The complicacy of the PPI network suggested that the tumor relapse of HCC is not driven by one or two AS events but by an integrated network. We further surveyed the ten hub proteins in the cluster with the highest score and found most of the hub proteins has been demonstrated to figure prominently in the development and progression of various tumor types. The top one hub protein AKT1, as one isoform of protein kinase B (PKB or AKT), has been reported to be involved in many tumor growth-related biology processes such as cell proliferation, apoptosis, growth, metabolism, and tumor angiogenesis and inflammatory cell infiltration by regulating mTOR, GSK3, BAD, p27KIP1, FoxO, and MDM2 signaling (Somanath et al., 2009; Hers et al., 2011; Fruman and Rommel, 2014; Mundi et al., 2016). Other two high-ranking hub proteins (HNRNPA1 and VEGFA) were also proved to play a vital role in tumor cell biology. HNRNPA1 has been reported overexpressed in many malignancies such as lung cancer, myeloma, leukemia, and Burkitt lymphoma (Roy et al., 2017). It accelerates cell cycle progression and aerobic glycolysis by activating telomerase to promote tumor growth, controls the anti-apoptotic signaling to enhance tumor maintenance and drug resistance, and advances the metastatic dissemination of cancer cells, all of which make HNRNPA1 promote various stages of cancer progression (Ting et al., 2009; Ko et al., 2014; Yu et al., 2015). VEGFA signaling played a crucial role in the progression of angiogenesis-related diseases, particularly in cancers; agents blocking VEGFA have been reported that could effectively inhibit tumor growth and metastatic spread (Claesson-Welsh and Welsh, 2013). The middle-ranking hub protein RHOA was well known as a signal mediator associated with multiple biological events such as cell polarity, cell morphology phenotypes, and migration, all of which are essential for progression of diverse malignancies (Nam et al., 2019). Another middle-ranking hub protein, RPS3, has been demonstrated to facilitate hepatocarcinogenesis through posttranscriptionally regulating SIRT1 (Zhao et al., 2019a). Moreover, hub protein MDM2, as E3 ubiquitin ligase, modulated tumor development and progression by forming an autoregulatory feedback loop with p53, which resulted in increased ubiquitin-mediated degradation of p53 (Konopleva et al., 2020). Other hub proteins SRSF1 and PTBP1 were considered splicing factors involved in cell proliferation, cell cycle progression, apoptosis, invasion, and migration, dysregulated expression of which has been validated to be associated with tumorigenesis and diminished immune response (Paz et al., 2021; Taniguchi et al., 2021). These previous research studies also validated the accuracy and convincement of our investigation. Similarly, in the regulatory splicing network, many AS events and SFs were associated with tumor cell biology. PCBP1, with the most interaction with the relapse-associated AS events in the core SFs, was identified as an intracellular immune checkpoint for maintaining the functions of effector T cells in tumor immunity and has been reported to inhibit HCC cell invasion by regulating the alternative splicing of CD44 (Zhang et al., 2010; Ansa-Addo et al., 2020). RBM25 acts as a tumor suppressor and regulator of MYC activity by controlling the splicing of the MYC inhibitor BIN1 (Ge et al., 2019). TIA1 and RBFOX2 were reportedly associated with tumor relapse or metastasis in malignancies (Hong et al., 2020; Mochizuki et al., 2021). Among the hub relapse-associated AS events in the splicing network, two AS events derived from one mRNA (ARAF-88922-AT and ARAF-88921-AT) were noted due to the most significance in the analysis. ARAF, as one isoform of the RAF family of kinases, has been proved to have an obligatory role in promoting MAPK activity and cell migration in a cell type-dependent manner, and ARAF mutations were identified in various tumor types which associated with resistance to RAF inhibitors (Mooz et al., 2014; ARAF Mutations Limit Response to RAF Dimer Inhibition, 2021). The results suggested that the dysregulated AS events of ARAF regulated by SFs may become a novel part of the mechanisms of tumor relapse in HCC, which needs further experimental investigation.

Subsequently, univariate Cox regression analysis was further performed, which identified 496 AS events derived from 377 mRNA to be prognostically significant. Based on the results, LASSO and multivariate Cox regression analysis were performed with tough screening standards and finally established a robust 16-gene AS signature for predicting tumor relapse in stage I–III HCC. According to the survival analysis, this novel relapse predictive AS signature displayed good performance in distinguishing the low-risk and high-risk groups in all of the training cohort, testing cohort, and entire cohort. The AUC values of different ROC curves at 1, 2, 3, and 5 year after surgery were all considerable in all the three cohorts, which indicated the great efficiency of the signature. Moreover, the results of subgroup analysis suggested that the relapse predictive signature was stable and has great performance in different conditions. After exploring the differentially expressed genes between the low- and high-risk groups, we found that these genes were significantly associated with immune-related pathways. Based on those results, the AS events involved in the signature may have important roles in tumor progression and regulate the immune microenvironment in HCC. The relevant genes of several AS events in this signature have been reported to play similar roles in human cancers by previous studies. For example, ACYP2 was reported to contribute to the malignant progression of glioma by promoting Ca^2+^ efflux and the subsequent activation of c-Myc and STAT3 signals (Li et al., 2020). Moreover, ACYP2 gene polymorphism was associated with the risk of cirrhosis developing into liver cancer, and high ACYP2 expression was associated with better overall survival in HCC, which indicated its important role in HCC progression (Zhao et al., 2019b). ANXA1 was highlighted as a biomarker in oncology, and manipulation of ANX1 in cancers can influence the metastatic behavior of the tumor cells by modulating inflammation, immune response, and angiogenesis (Delorme et al., 2021). COMT was considered a tumor suppressor that is associated with anticarcinogenesis, antiproliferation, pro-apoptosis, anti-angiogenesis, and anti-inflammation (Bastos et al., 2017). RNASEH2C was reported as a metastasis susceptibility gene and modulator of T cell-mediated immune response in breast cancer (Deasy et al., 2019). SH2D4A was identified as a chromosome 8p tumor suppressor and positively correlated with effector and regulatory T cell infiltration by blocking IL-6 signaling in HCC, which implies its crucial role in HCC (Ploeger et al., 2016). Furthermore, our ssGSEA analysis demonstrated that the local infiltration fractions of several important immune cells, including CD8 + T cells, DCs, macrophages, neutrophils, pDCs, Th cells, and TILs, were significantly higher in the lower-risk group than in the high-risk group. Similarly, the local immune functions of CCR, checkpoint, cytolytic activity, HLA, inflammation-promoting, parainflammation, T cell co-stimulation, type I IFN response, and type II IFN response were also more activated in the low-risk group than in the high-risk group. Moreover, ESTIMATE analysis revealed that the low-risk group exhibited significantly higher ESTIMATE score, immune score, and stromal score, which further confirmed the ssGSEA results. Finally, unsupervised consensus clustering was further conducted based on the AS events involved in the relapse predictive signature, and the entire cohort of the included stage I–III HCC patients was divided into four molecular subtype clusters. The molecular subtype clustering exhibited good performance in distinguishing the different relapse patterns and local immune infiltration status between diverse clusters, which further verified the power of the signature.

## Conclusion

In summary, our study first provides an overview of the relapse-associated AS events in stage I–III HCC and further constructed a novel AS signature that robustly stratifies tumor relapse risk, indicates immune activity, and facilitates identifying molecular subtypes in stage I–III HCC. The results of the study facilitate the deep mining of the mechanisms associated with tumor relapse and tumor immunity and the development of novel individualized treatment targets for HCC.

## Data Availability

The datasets presented in this study can be found in online repositories. The names of the repository/repositories and accession number(s) can be found in the article/Supplementary Material.

## Supplementary material

SUPPLEMENTARY TABLE S1 Information of the PPI network.

SUPPLEMENTARY TABLE S2 Information of SF–AS correlation network.

SUPPLEMENTARY FIGURE S1 Functional enrichment analysis of the relevant genes of the relapse-associated AS events. Top ten significant enriched terms in the three GO categories (BP, CC, and MF) were shown. Abbreviations: BP, biological processes; CC, cellular components; GO, Gene Ontology; MF, molecular functions.

SUPPLEMENTARY FIGURE S2 Forest plots depicting the results of Cox regression analysis. **(A)** Univariate Cox regression analysis; **(B)** multivariate Cox regression analysis.

## Glossary

HCC: Hepatocellular carcinoma
AS: Alternative splicing
SFs: Splicing factors
TCGA: The Cancer Genome Atlas
LIHC: Liver hepatocellular carcinoma
UCSC: University of California Santa Cruz
AA: Alternate acceptor site
AD: Alternate donor site
AP: Alternate promoter
AT: Alternate terminator
ES: Exon skip
ME: Mutually exclusive exons
RI: Retained intron
PSI: Percent-spliced-in
SF: Splicing factor
RFS: Relapse-free survival
PSM: Propensity score matching
GO: Gene Ontology
BP: Biological processes
CC: Cellular components
MF: Molecular functions
PPI: Protein–protein interaction
cor: Correlation coefficient
ROC: Receiver operating characteristic
KEGG: Kyoto Encyclopedia of Genes and Genomes
ssGSEA: Single-sample gene set enrichment analysis
DCs: Dendritic cells
pDCs: Plasmacytoid DCs
Th: T helper
TILs: Tumor-infiltrating lymphocytes
CCR: Chemokine receptors
HLA: Human lymphocyte histocompatibility antigen
IFN: Interferon
AJCC: American Joint Committee on Cancer
PKB: Protein kinase B
