# Supplementary figures and images for: A novel 16-gene alternative mRNA splicing signature predicts tumor relapse and indicates immune activity in stage I–III hepatocellular carcinoma
Source: Front Pharmacol. 2022 Sep 6;13:939912. doi: 10.3389/fphar.2022.939912 (PMC9485890; doi:10.3389/fphar.2022.939912)

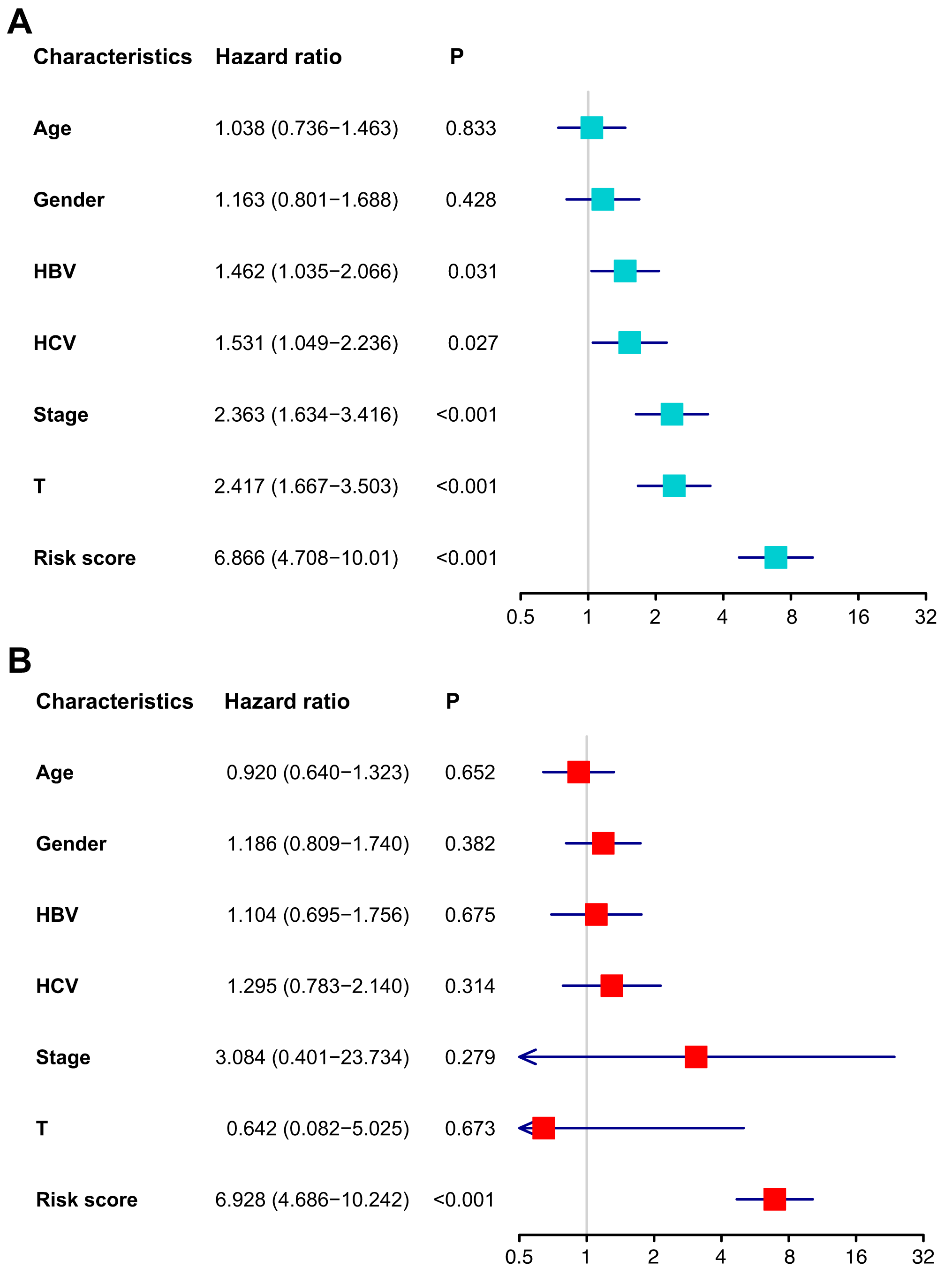

Supplement: Supplementary file 2 [file Image2.TIF]

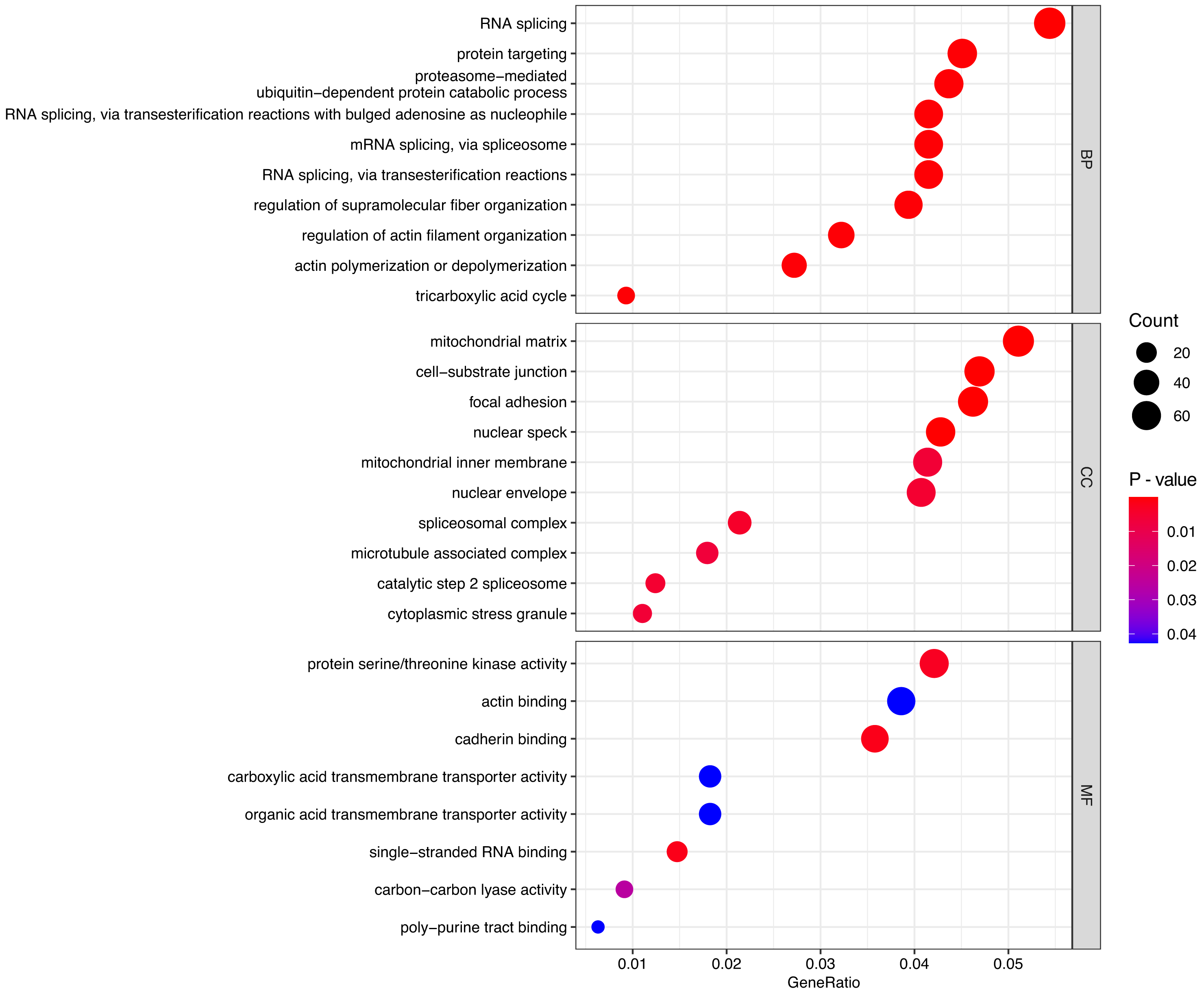

Supplement: Supplementary file 3 [file Image1.TIF]
